# Effects of sediment flushing operations versus natural floods on Chinook salmon survival

**DOI:** 10.1038/s41598-022-19294-2

**Published:** 2022-09-12

**Authors:** Manisha Panthi, Aaron A. Lee, Sudesh Dahal, Amgad Omer, Mário J. Franca, Alessandra Crosato

**Affiliations:** 1 Department of Water Resources and Ecosystem, IHE Delft Institute for Water Education, Westvest 7, 2611 AX Delft, The Netherlands; 2 Utah Water Research Laboratory, Department of Civil and Environmental Engineering, Utah State University, 1600 Canyon Rd, Logan, UT 84321 USA; 3 Natural Systems Design, 127 E 1st. St., Port Angeles, WA 98362 USA; 4 Laboratory of Hydraulics, Hydrology and Glaciology, ETH Zürich, Hönggerbergring 26, 8093 Zurich, Switzerland; 5 Rivers-Reservoirs Dynamics and Morphology, Deltares, Boussineqweg 1, 2629 HV Delft, The Netherlands; 6 Karlsruhe Institute of Technology, Engesserstraße 22, Geb. 10.83 - Raum 108, 76131 Karlsruhe, Germany; 7 Department of Hydraulic Engineering, Faculty of Civil Engineering and Geosciences, Delft University of Technology, Delft, The Netherlands

**Keywords:** Environmental impact, Ecology

## Abstract

Flushing is a common measure to manage and reduce the amount of sediment stored in reservoirs. However, the sudden release of large volumes of sediment abruptly increases the suspended solids concentration and alters the riverbed composition. Similar effects can be produced also by natural flood events. Do flushing operations have more detrimental impacts than natural floods? To answer this question, we investigated the impact of flushing on the survival of the Chinook salmon (*Oncorhynchus tshawytscha*) in the Sandy River (OR, USA), assuming that sediment is flushed from hypothetical bottom gates of the, now decommissioned, Marmot Dam. The effects of several flushing scenarios are analyzed with a 2D morphodynamic model, together with habitat suitability curves and stress indicators. The results show that attention has to be paid to duration: the shorter the flushing operation, the lesser the stresses on fish survival and spawning habitats. Flushing causes high stress to salmon eggs and larvae, due to unbearable levels of suspended sediment concentrations. It also decreases the areas usable for spawning due to fine-sediment deposition, with up to 95% loss at peak flow. Without the dam, the corresponding natural flood event would produce similar effects, with up to 93% loss. The study shows that well-planned flushing operations could mimic a natural impact, but only partly. In the long-term, larger losses of spawning grounds can be expected, since the removal of fine sediment with the release of clear water from the reservoir is a lengthy process that may be undesirable due to water storage reduction.

## Introduction

Reservoirs trap sediment transported by rivers, with coarser material settling first, near the reservoir entrance, and finer sediment reaching further areas closer to the dam^1^. Progressive deposition of sediment reduces the water storage capacity and impacts reservoir and dam operations^2–4^. Flushing operations are a way to remove part of the deposited sediment, particularly the finest component that accumulates near the dam^5^. However, the rapid release of large quantities of water and sediment produces sudden alterations in flow velocity, water depth^6^ and temperature^7^, together with an abrupt increase of the concentration of suspended solids^2,8–12^ in the downstream river. These sudden alterations highly impact the river ecology^13–16^, also because they often produce persisting changes in bed composition and morphology^17–19^.

The release of fine sediment affects fish and macroinvertebrates in several ways. High concentrations of suspended sediment have acute/short-term effects on respiratory organs, which can be lethal^20^, whereas the attenuation of light penetration produces negative effects on fishes and other forms of life, and hence their growth and development^21^. The release of reservoir water often results in a change in water temperature^7^ and this can lead to a behavioral drift and alter the distribution of benthos, as well as increase the mortality of invertebrates^22^. In addition, the deposition of fine sediment on the riverbed may: (i) bury and suffocate benthos, laid eggs, larvae and fry (acute/short-term effect)^23,24^; (ii) deteriorate spawning grounds (mid-to-long-term effect)^25–27^; and (iii) clog the hyporheic region of the bed hindering fluxes of oxygen and metabolic waste, as well as groundwater-surface water exchanges (mid-to-long-term effect)^28^.

Quantifying the effects of flushing operations on biota requires the assessment of both acute and long-term impacts^29^. However, most studies focus either on the effects of high suspended solids concentrations^10,30,31^ or on riverine habitat deterioration^32,33^. Empirical relations have been developed to quantify the effects of high levels of suspended sediment concentration on fish^20,34,35^, whereas habitat suitability curves can be used to quantify the impact of fine sediment on spawning grounds^25,29,36,37^.

Natural floods might have similar acute and long-term effects, because they also present a sudden increase in flow velocity and sediment transport, particularly in mountain streams^38–41^ and near the vicinity of urban areas, due to heavy rainfall^42,43^. However, in general, instream biota have adapted to the specific hydrology and sediment transport regimes of their river system, so natural floods may not threaten the long-term survival of fish and invertebrate species^38,40,41,44–47^. What is then the difference between natural floods and flushing operations? Can reservoir-flushing operations be designed with reduced acute and long-term effects on fish?

To answer these questions, this research evaluates and compares the effects of both flushing operations and the corresponding natural flood on the survival and spawning habitats of Chinook salmon (*Oncorhynchus tshawytscha*) in the Sandy River (OR, USA). The study considers both the severity of stress caused by high sediment concentrations (acute short-term effects) and the decrease of spawning habitat due to fine sediment deposition (medium to long-term effects). A key assumption is that the Marmot Dam, built on the Sandy River in 1913 and decommissioned in 2007, is operational and provided by bottom gates for sediment flushing. The releases of water and sediment volumes from the reservoir are derived by simulating the opening of the hypothetical bottom gates of the dam using a two-dimensional (2D) morphodynamic model, here called the Reservoir model. The Marmot Dam is assumed absent for the quantification of the flow discharges and the sediment transport rates during the natural flood. The changes in hydraulic and sediment characteristics in the downstream river reach are computed with another 2D model, here called the River model. The results are post-processed to estimate the potential impacts of the considered events on Chinook salmon.

### Case study area

**Figure 1:**
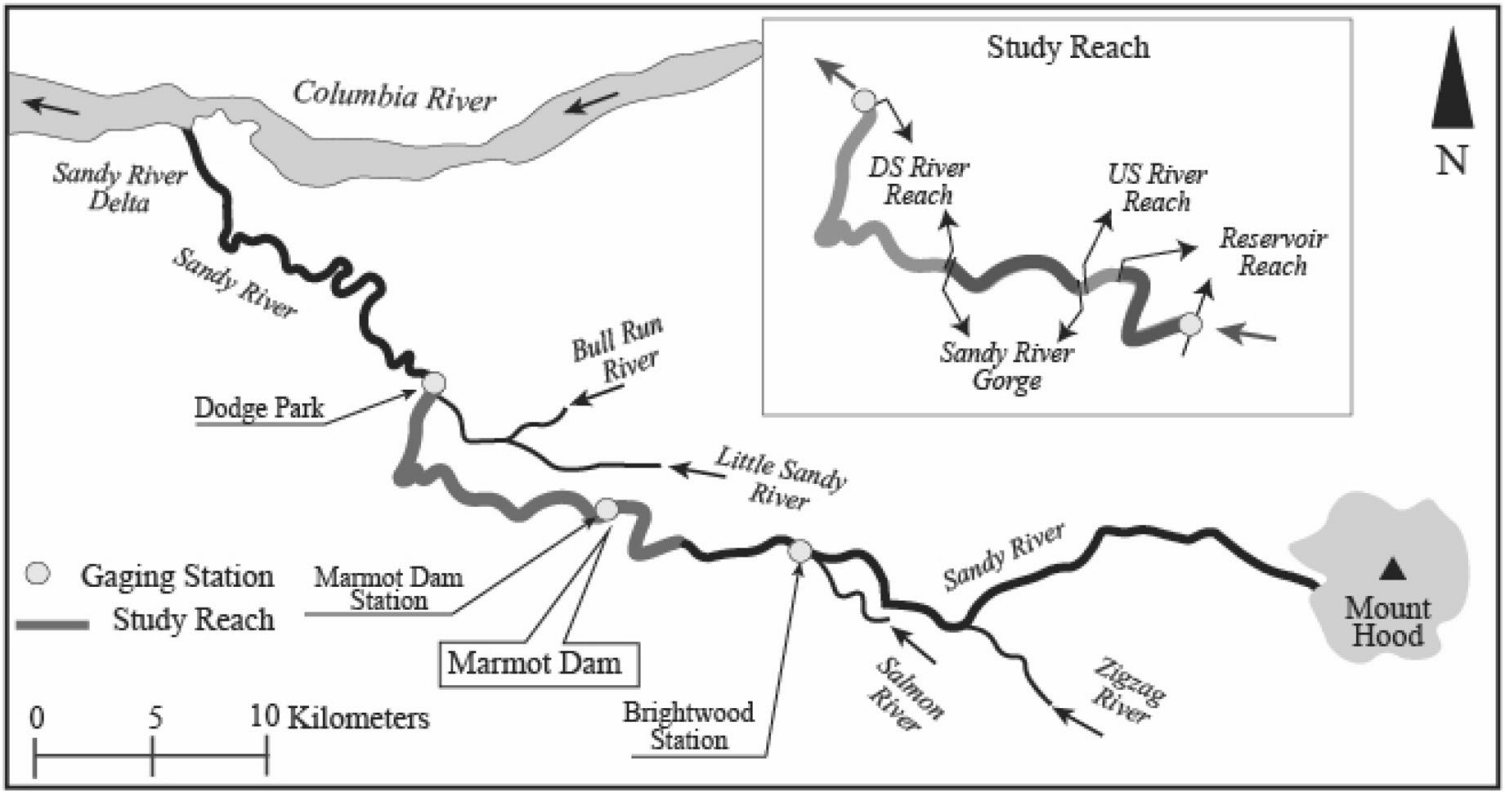
Location of Marmot Dam and study area showing the tributaries and gauging stations (Adapted from Major et al.^9^, Credit: Department of Interior/ USGS) created using Adobe Illustrator 2021: https://www.adobe.com/products/illustrator.html, U/S indicates “upstream” and D/S “downstream”.

In 1913, Portland General Electric (PGE) constructed the Marmot Dam across the Sandy River, 48 km upstream of the confluence with the Columbia River, as a wooden crib dam for hydropower generation (Fig. 1). The dam was later upgraded to a 15 m high concrete structure with an overflow spillway crest^48^ without bottom gates, which restricted bedload transport downstream. It was finally removed in 2007, when the anticipated cost of upgrading for the licensing requirement had become higher than the benefits.

At the time of dam removal, the reservoir was filled by 750,000 m^3^ of sediment, ranging from silt to boulders subdivided into layers of different compositions^9,49^. The upper layers comprised coarser sediment, mainly gravel and cobbles. While the lower layers contain fine sediment, ranging from clay to sand, but dominated by sand. A coarser layer underneath was the original riverbed. The dam removal was facilitated by the construction of a temporary cofferdam which diverted the flow of Sandy River bypassing the concrete dam; this cofferdam was later breached in a controlled way.

The Sandy River flows from the western part of the Mt. Hood to the Columbia River. Its major tributaries are the Zigzag River, the Salmon River, the Little Sandy River, draining from Mt. Hood, and the Bull Run River draining from the Bull Run Lake. The combined basin area is 1300 km^3^, with an altitude ranging from 3428 m at Mt. Hood, to 3 m a. s. l.^9^ at the confluence with the Columbia River (Fig. 1). The gradient of the Sandy River gradually reduces from its steep mountain parts, where it transports cobbles and boulders, to its confluence, where, as low-gradient sand-bed river, it forms an inland delta. The river is fed by rainfalls and spring melts from snowpack^50^. Three-fourths of the annual precipitation falls in the period October–March, with the highest amounts occurring in November, December and January^51^.

The study area extends from 3.5 km upstream to 18 km downstream of the (ex) Marmot Dam where the Sandy River meets the Bull Run River. At the Marmot Dam location, the river has a mild slope and presents a wider floodplain but 5 km downstream of the dam the river is confined and steep. Here the river presents rock outcrops and its bed is only partly alluvial. This river reach, known as the Sandy River Gorge, extends for 6.5 km. At the exit of the gorge, the river becomes unconfined with a mild slope, forming meanders for another 9.4 km. These reaches of river have different characteristics altering the flow depth, velocity and bed material composition, hence the use of these areas by different life stages of Chinook salmon differs with time of the year altering the consequences spatially and temporally.

In 1964, after a flood with a discharge peak of 2400 m^3^/s, the highest since 1910, parts of the Sandy River and its tributaries were artificially straightened, their banks protected by rock berms and large obstructions caused by boulders and woody debris were removed^52^. After the removal of the Marmot Dam (2007) the river runs freely without any discharge regulation, providing extended habitat for existing biotas.

The abundance of hydrodynamic and morphological data before and after the removal of the Marmot Dam is the reason behind the choice of this case study, since it allowed the construction of two modeling tools simulating the erosion of the reservoir deposit during flushing operations^5^ and the transport and deposition of the released sediment along the Sandy River^32,53^.

### Chinook salmon (*Oncorhynchus tshawytscha*)

Two Pacific salmon species find their spawning habitats in the Sandy River^52^: Chinook salmon, both spring-run and fall-run, and coho salmon. Chinook is the largest Pacific salmon species, reaching a weight between 6 and 23 kg^54^. As most of Pacific salmon, the adults return to their natal gravel-bed streams from the ocean to spawn. The alevins emerge from the eggs and live within the gravel until they are large enough (fry) and start their migration towards deeper waters to finally reach the ocean where they grow into adults^55^. With a water temperature of 11 °C, Chinook salmon eggs hatch in roughly 47 days, whereas the alevins need 84 days to absorb their yolk sacs and become fry^56,57^.

The spawning periods of fall and spring Chinook salmon in the Sandy River are September-December and September–October, respectively^58^. This means that fry appear in December-March, approximately four months after eggs are laid.

The spawning grounds are especially found upstream of the ex-Marmot Dam and downstream of the Dodge Park (Fig. 1)^59^. The requirements regarding the physical properties (flow, velocity, depth) and water quality (temperature, dissolved oxygen, etc.^60,61^) must be met for successful salmon spawning^37,62^. Other important requirements are:

1. Adult fish must arrive healthy at spawning grounds. This requires river continuity and that sub-lethal or lethal stress conditions are not met during migration and spawning.
2. Spawning fish must be able to construct a nest. This requires appropriate gravel size at spawning areas.
3. The nest must not be scoured or suffocated during egg incubation. This requires a strongly limited presence of fine sediment among gravel.

## Materials and methods

### General approach

Our investigation focuses on Chinook salmon, considering two main effects of reservoir flushing and natural flood events: the effects of acute stress conditions imposed during these extreme occurrences and the effects of fine sediment deposition in the spawning areas.

Two distinct two-dimensional morphodynamic models are used: the first one, the Reservoir Model, is used to route the water and sediment through the reservoir, generating water and sediment fluxes to the river downstream of the dam. It is an extended version of the model of Dahal et al.^5^. The second one, the River Model, represents the Sandy River from the downstream of Marmot Dam location to the confluence with the Bull Run River. It is an extension of the model developed by Lee^32^.

The two models were developed using the open-source Delft3D code version 4.03 (https://www.deltares.nl/en/software/delft3d-4-suite/) considering sediment ranging from sand to cobbles, neglecting thus the finest components of the deposited material that are transported in suspension: fine sand, silt, and clay. Considering that this study focuses on fine sediment processes, both models had to be extended.

**Figure 2:**
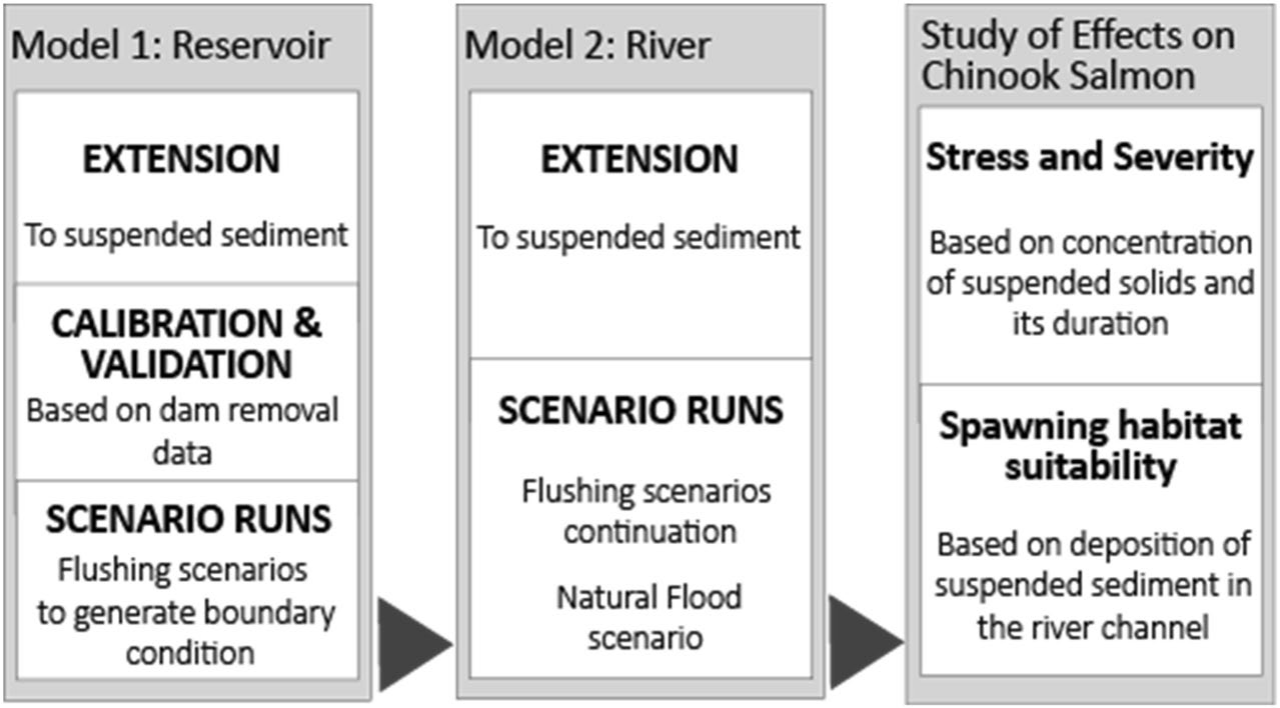
General approach of the study and workflow direction developed using Adobe Illustrator 2021 (https://www.adobe.com/products/illustrator.html).

The extended Reservoir Model is calibrated and validated, whereas lack of data on fine sediment deposition and transport rates along the Sandy River make calibration and validation of the extended River model impossible. For this, the runs include a sensitivity analysis on the effects of changing the size of suspended sediment, represented by its fall velocity, with the idea of covering a reasonable range of plausible scenarios. The results of the River model indicating the effects on water flow and sediment of the flushing scenarios and of the natural flood are then analyzed by means of stress and severity indices and suitability curves distinguishing the different life stages of salmon and its spawning habitat. Figure 2 schematizes the approach and the work flow.

### Modeling tools

Delft3D is an open-source software used to simulate non-steady flow and transport phenomena in rivers, coasts and estuaries. It allows simulating 2D and 3D hydrodynamic and transport processes of sediment mixtures^5,63–65^. The use of both cohesive and non-cohesive sediment of different sizes is possible, and makes this tool useful for investigating sediment transport processes in different contexts^66^.

**Figure 3:**
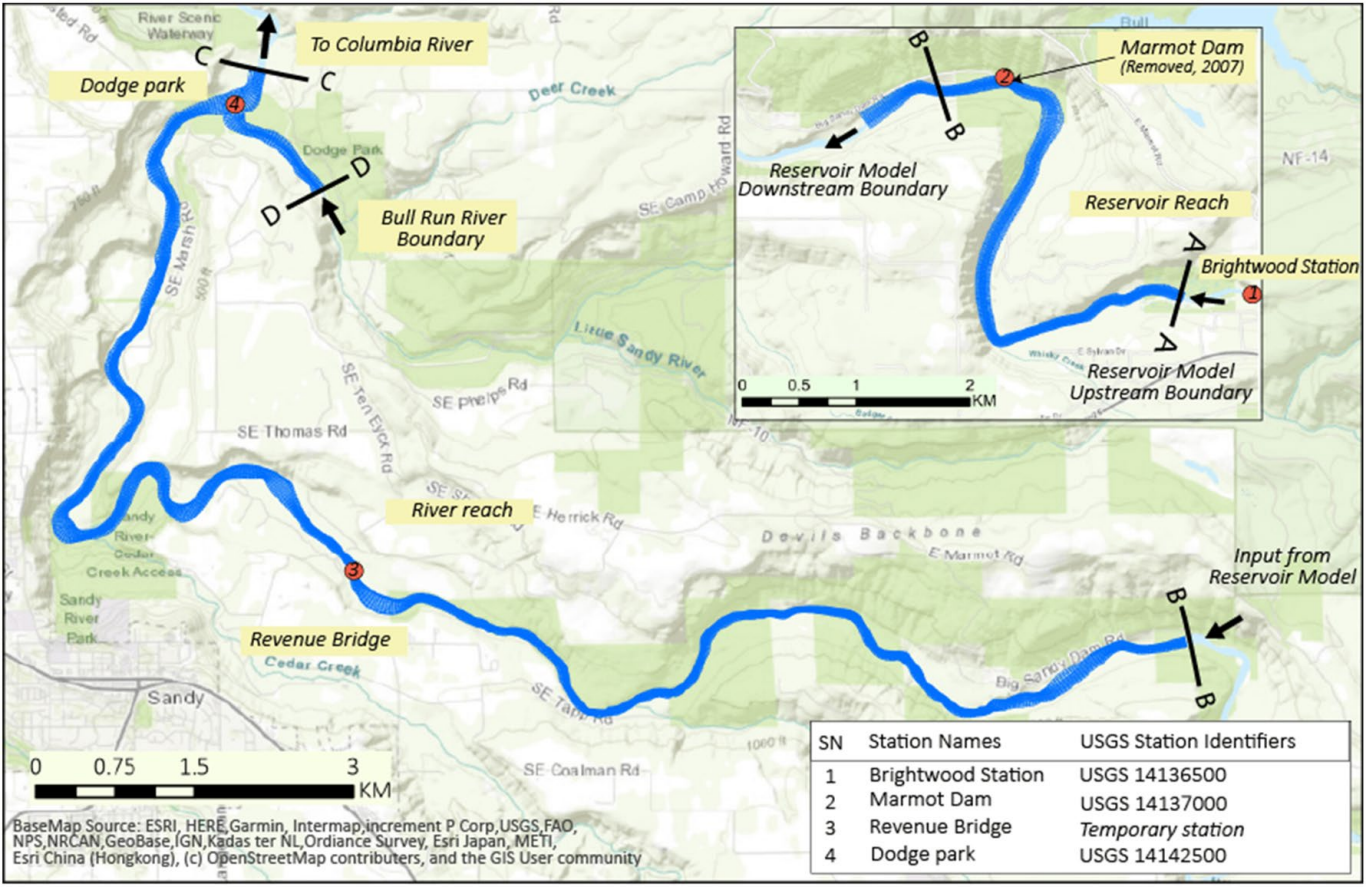
Domain of the Reservoir and River models. The boundaries are indicated by cross-sections identified by letters. Along the Sandy River the letters are in alphabetic order from upstream to downstream. Note that the boundary B defines the junction between the two models. The inflow boundary of the Bull Run River is indicated by the letter “D”. The numbered red dots indicate the USGS gauging stations. Names and identifiers are listed in the right-below corner. The figure is developed using QGIS 3.16.16 (https://qgis.org/en/site/index.html) and edited in Adobe Illustrator 2021 (https://www.adobe.com/products/illustrator.html).

The extended models used in this study are two-dimensional (2D) and solve depth-averaged shallow-water equations. The transport of suspended sediment is computed by means of 2D advection–diffusion equations coupled with a sediment entrainment and a sediment deposition equation. The sediment entrainment rate is assumed to be proportional to the difference between the local bed shear stress and its critical value for bed erosion, following the Krone and Partheniades approach^67,68^, multiplied by a coefficient, here named erosion coefficient. The deposition rate is obtained by multiplying the fall velocity of the sediment particles with the local depth-averaged sediment concentration^69^. Transport capacity formulas are used for the computation of bedload rates. The presence of several grain sizes requires considering the hiding of the smaller particles provided by the presence of the larger particles and the exposure of the latter being surrounded by smaller particles. However, the effect of hiding and exposure is not accounted in Delft 3D in the combination bedload/suspended load. This might overestimate the transport rates of the smallest bed-load particles and underestimate the largest particles. The computation of bed level change is based on sediment balances. For fine sediment travelling in suspension, bed level changes are given by the difference between sediment deposition and sediment entrainment rates. For bedload, the sediment balances follow Exner’s approach^70^. The effects of transverse slope on bedload direction, important for 2D bed topography changes, are accounted for according to Ikeda^71^, and the effects of longitudinal slope according to Bagnold^72^. The domains of the models and their computational grids are shown in Fig. 3.

### Model 1: Reservoir model

The Reservoir model comprises a spatial domain that covers the Marmot Dam and its reservoir area (3.5 km long), with the boundaries being located 1 km upstream of the reservoir influence zone and 1.4 km downstream of the Marmot Dam, schematized with a curvilinear grid (Fig. 3).

In the Delft3D model, the strata in the reservoir sediment deposit can be distinguished by sediment composition, extension, and thickness. Based on the granulometric analyses of the sediment collected from the different strata^49^ in the reservoir (refer Fig. 10 of the report from Stillwater Sciences^71^), the extended model includes three sediment fractions: fine sediment (several sizes from sand to clay, depending on scenario), gravel (D_50_ = 20 mm), and cobbles (D_50_ = 100 mm). The distinction in fractions is needed to correctly describe the processes of sediment transport, bed erosion and deposition along the river. The composition of units^5,73^ are selected as, Unit 1 imposed with a ratio 25:35:40 for fine sediment, gravel and cobbles, whereas Unit 2 and of the pre-dam riverbed have ratios 85:15:0 and 25:30:45, respectively. The use of different units and composition of strata is needed to correctly simulate sediment removal from the reservoir during the flushing operation. A cofferdam and wash load layer are also added to facilitate breaching. These percentages of sediment composition are derived from a range of percentages, since the sediment composition depends on location (data from Squier Associates^49^). Bedload is computed using the capacity formula of Aschida and Michiue^74^.

The time series of the discharge measured at the Marmot Dam station (located 400 m upstream of Marmot Dam before dam removal and shifted downstream of Dam after removal) allows constructing the inflow hydrograph, whereas the sediment input at the upstream boundary is generated using a regression analysis from the available sediment measurement data at the Brightwood station.

To properly reproduce the fine sediment input to the downstream river, a wash load layer, is added in the last 500 m of the reservoir to represent the most recent suspended sediment deposits located in the downstream part of the reservoir, at the time of cofferdam breaching.

The extended Reservoir model is calibrated and validated for sediment erosion after dam removal to establish the properties of fine sediment. During the calibration process, the fall velocity and the erosion coefficient parameters, among others are tuned, to reproduce the field data, of suspended solids concentration measured during and after the Marmot Dam removal, assuming this operation is an extreme case of sediment flushing. The calibration runs cover a period of 5 days, which involves the dynamic process of cofferdam breaching. The cofferdam breaching process is modeled as a notch that is cumulatively eroded by flowing water. Since the outflow to the downstream river reach is a function of the cofferdam breach process, the measured water discharge record at the Marmot Dam station is used as a calibration metric. The concentration of the wash load layer is calibrated on suspended sediment concentration measurements, peaking at 49 g/m^3^, during dam removal^9^.

Validation of the extended model is based on measured data of sediment concentration and reservoir bed erosion. These data were collected in the three months that followed dam removal, during which 45% of the reservoir sediment was eroded. Information on the erosion process is acquired from the USGS report^9^ (https://pubs.usgs.gov/pp/1792/) and from the surveys conducted by USGS, David Evans and PGE*.* Detailed information on the extended model is provided by Panthi^75^.

**Figure 4:**
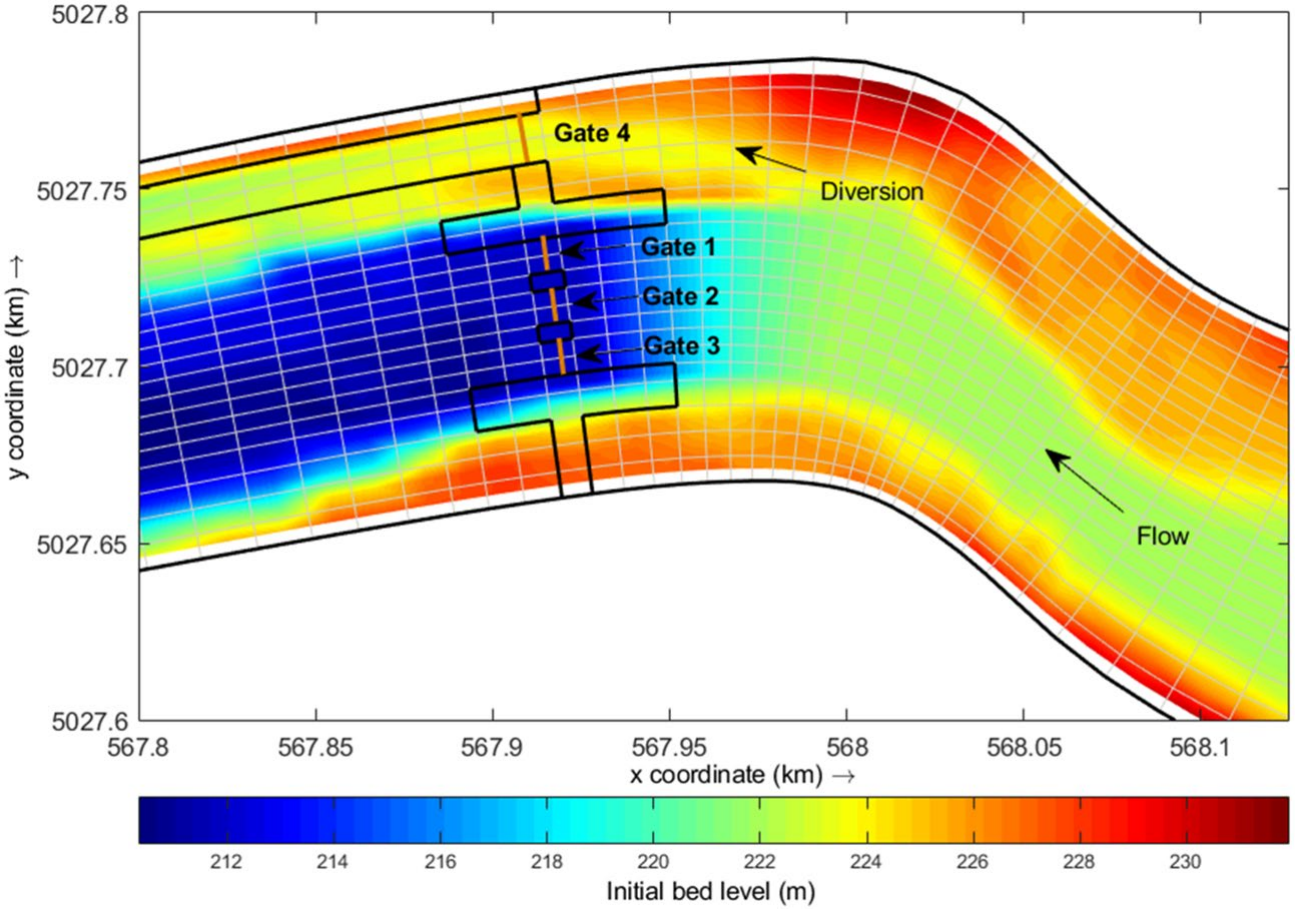
Reservoir model at the location of the Marmot Dam with hypothetical gate openings, with three gates for flushing and one for water diversion use.

For the flushing scenarios, a virtual Marmot Dam consisting of bottom gates is introduced at the old dam location (Fig. 4). A real-time control (RTC) mechanism is used in compliance with the model to simulate the openings of the gates^5^.

### Model 2: River model

The Sandy River model covers 18 km from the Marmot Dam to the confluence of the Bull Run River (Fig. 3). The model grid comprises 1133 × 61 curvilinear cells.

Lee^32^ derived the initial riverbed topography from the LiDAR survey 2007^32^, but this does not comprise the submerged portions of the channel bed. An initial run (spin-up) was thus executed to compute a complete realistic 2D riverbed topography and sediment composition. The spin-up simulation started with a transversally plane-bed in the submerged portion, composed of sand, gravel, and cobbles^32^ with sizes of 0.3, 22 and 100 mm, respectively. The morphological evolution of the riverbed was obtained by imposing a schematic discharge time series with no sediment input, due to the presence of the dam, with a time step of 0.05 min. The sediment transport rate was computed with the transport formula of Meyer-Peter-Muller^76^, applicable for bedload transport in gravel-bedded streams. The result of the spin-up run was then compared with the morphological features that are visible from aerial imagery, such as extension and location of sediment deposits and the deep areas that are evident at low-flow conditions. Model calibration is based on this comparison, since no measured data on bed topography are available for the submerged part of the river channel.

Information regarding the sediment composition of the Sandy River before dam removal is limited to pebble counts. Field investigations found bed armoring downstream of the Marmot Dam with an average grain size of the bed material of 100 mm^8,73,77,78^, whereas the percentage of fines was estimated from visual valuation^32^. This means that there is no way to quantitively check the model results in terms of sediment composition.

The River model^32^ is here extended and the spin-up run re-done to include the suspended-sediment processes. The transport of coarse material is computed with the transport formula of Meyer-Peter and Müller^76^, whereas the transport of fine sediment is computed by means of advection–diffusion equations coupled to sediment entrainment and deposition formulations. The characteristics of the sediment-related variables in the extended model are listed in Table 1. Note that the adopted sediment characteristics are those obtained from the calibration of the extended Reservoir model. The initial bed composition is derived from the characteristics of the strata provided by Squier Associates^49^. Morphological calibration of the extended River model is not possible due to lack of measured data on bed topography, bed composition and deposition of fine sediment in the river downstream of the Marmot Dam. This means that some scenarios have to include different sediment characteristics to cover a wide range of possibilities.

**Table 1:** Initial bed composition, sediment characteristics and sediment transport formula in the spin-up run of the River model computing the initial state of the river.

Bedload characteristics		Suspended load characteristics	
Gravel (D_50_)	20 mm	Fall velocity	0.036 m/s
Cobble (D_50_)	100 mm	Erosion coefficient	4.17*10^−3^ kg/m^2^/s
Transport Formula	M-P* Muller^76^	Critical shear stress	1 N/m^2^

Where D_50_ is the median diameter of bedload material.

*M-P = Meyer-Peter.

### Scenarios

A natural flood described by the discharge hydrograph of December 2007, with a sharp rising limb and a gentler falling limb, typical of Sandy River floods, is selected as a reference for the flushing and the natural flood scenarios (see Supplementary Fig. A1). Based on the flow frequency statistics provided by Major et al.^9^, this flood has a return time of less than two years. The boundary conditions of the Reservoir model are the measured daily discharge time series and the sediment inflow derived from the data measured at the Brightwood station. The input of suspended solids is derived using a relation between discharge and sediment load generated from a regression analysis of the available sediment concentration data from the same station.

The flushing scenarios are chosen based on the timing of gate opening during the peaking of the flood wave, with only Gate 2 (width: 9.72 m; height: 3 m) in operation (Fig. 4). This pattern of gate opening is considered the most effective one^5^, since it allows flushing higher volumes of sediment in a shorter period.

In the corresponding natural flood scenario, the Sandy River is assumed to be freely flowing without the dam. This means that in this case the Reservoir model is not used to generate the discharge and sediment inputs to the River model. The input of fine sediment to the river is computed from the regression law derived for the Brightwood station. The input of coarse sediment is computed using the selected transport formula. All scenarios are listed in Table 2.

**Table 2:** Model scenarios.The River model also includes the additional natural flood scenario.

Scenario number	Reservoir model		River model
	Input: discharge & sediment load		Input = Output from reservoir model
1	RF_20	Opening at 20% of peak (Q_in_ = 48 m^3^/s)	RF_20
2	RF_50	Opening at 50% of peak (Q_in_ = 120 m^3^/s)	RF_50
3	RF_80	Opening at 80% of peak (Q_in_ = 192 m^3^/s)	RF_80
4	No dam		Natural flood (Upstream boundary as described in Fig. A1)

The effects of different suspended sediment sizes are studied separately considering three fall velocities representing the characteristics of clay, silt, and medium sand (see Supplementary Table A1). The input of water and sediment to the river for these three scenarios corresponds to reservoir flushing with gate opening at 80% of peak flow (moment of gate opening is indicated in Supplementary Fig. A1).

### The impact of suspended solids concentration: stress and severity on salmonids

High suspended sediment concentration in the water column causes acute effects on aquatic biota. These short-term effects are not accounted while defining the suitability of habitats, but they can create an unbearable environment and cause behavioral problems, physical damage and even death to fish and invertebrates^20,24,30,31,34,35^. The exposure to suspended solids is detrimental to salmon species too and depends on concentration and duration of exposure^34,79^. The severity scale developed by Newcombe and Jensen^35^ is here used to study the acute impact on salmonids caused by high suspended sediment concentrations during the flushing operation or by the flood event. The severity scale is given as a function of concentration of suspended sediment (SSC) and duration (D).

The severity scale of Newcombe and Jensen are based on regression lines fitting literature data on various salmon species, considering suspended sediment with particle size up to 250 µm^35^. The scale relates to the effects of SSC on individual fish and does not account the fish preconditions nor any other factors causing unsuitable environment, e.g. rise in water temperature or decrease in dissolved oxygen. Considering the high level of uncertainty, also related to the fact that the data are from differing river environments, the severity index should be used to compare situations rather than to quantitatively predict the effect of SSC on a specific fish species in a specific river. Notwithstanding this, the choice of using this severity index lies in its simplicity of application and in the scope of the work, aiming at comparing the effects of flushing operation and flood events.

Newcombe and Jensen^35^ expressed severity on a scale of 0 to 14, distinguishing the effects in behavioral (0–4), sub-lethal (5–8) and lethal (9–14) for each life stage.

Adult salmonids

1 $$\begin{array}{*{20}c} {SEV = 1.6814 + 0.4769 \left( {\log_{e} D} \right) + 0.7565\left( {\log_{e} SSC} \right)} \\ \end{array}$$

Juvenile salmonids

2 $$\begin{array}{*{20}c} {SEV = 0.7262 + 0.7034 \left( {\log_{e} D} \right) + 0.7144\left( {\log_{e} SSC} \right)} \\ \end{array}$$

Eggs and larvae of salmonids

3 $$\begin{array}{*{20}c} {SEV = 3.7466 + 1.0946 \left( {\log_{e} D} \right) + 0.3117\left( {\log_{e} SSC} \right)} \\ \end{array}$$

where, D is the duration of exposure in hours and SSC is the suspended sediment concentration in mg/l.

The severity indexes are here calculated using three different metrics:

i. The concentration of suspended sediment is plotted against the duration in which it is exceeded, which creates a concentration duration curve of the flushing operation. The value of the concentration that is exceeded for 50% of the time is then multiplied by total duration of the flushing operation to calculate the representative value of severity^30^.
ii. The time series of severity is computed for the different life stages based on the duration curves of suspended sediment concentration for different locations along the river reach.
iii. The acceptable duration of exposure is compared with the actual duration for different concentration values^35^.

### Fine sediment deposition: impacts on spawning areas

Deposition of fine sediment on spawning grounds with laid eggs can cause mortality and sub-lethal effects to eggs and emerging larvae, creating an acute impact on salmon^80^. Moreover, the deposition alters the composition of the riverbed, which may further result in unsuitability of the area as spawning ground, which might be a persisting, and thus long-term effect.

Habitat suitability index (HSI) models are developed to categorize and quantify the ability of defined areas to meet the physical requirements of specific habitats. They relate the values of each physical variable describing the local aquatic environment^81,82^, e.g. flow velocity, water depth, bed composition and water temperature, to its suitability for a specific species or populations of fish or invertebrates^25,28,29,36,37,83^. Considering the strongly differing requirements, distinct indices are derived for eggs, larvae, juveniles and adults of the same species^35^. Each HSI is normally based on the statistical analysis of scarce field data^84^, for instance local fish counts versus the value of a specific physical variable. It may thus present a large degree of uncertainty due to the weak or even inconsistent statistical relations that are often found^85^. Nevertheless, habitat suitability indexes are commonly used to assess the distribution of specific species, as well as to objectively translate the physical alterations caused by external factors into effects on habitats^86^.

This study aims at highlighting the changes in spawning habitat suitability caused by flushing operations or by the corresponding flood event. Being specific for Chinook salmon, the study adopts the HSI model developed by Raleigh et al*.*^37^ based on a large number of field and laboratory data. The analysis is based on three variables: water depth, flow velocity and percentage of fines in the riverbed. The index is scaled as a real number from 0 to 1, where 0 means unsuitable and 1 is an optimal condition. HSI is computed for different life stages. The HSI is then calculated as the product of the suitability indexes of the three variables as

4 $$\begin{array}{*{20}c} {HSI = SI_{1} * SI_{2} * SI_{3} } \\ \end{array}$$

The total suitable area for spawning in the considered river reach is finally calculated by applying the total weighted usable area (WUA) approach^29^ in which the suitable area is expressed as a percentage of the total surface area (wet area) of the considered reach:

5 $$\begin{array}{*{20}c} {WUA = \sum A_{i} HSI_{i} } \\ \end{array}$$

where, A_i_ is area of the grid cell and HSI_i_ is the value of the HSI value for the same cell.

## Results

### Reservoir model: calibration and validation

**Figure 5:**
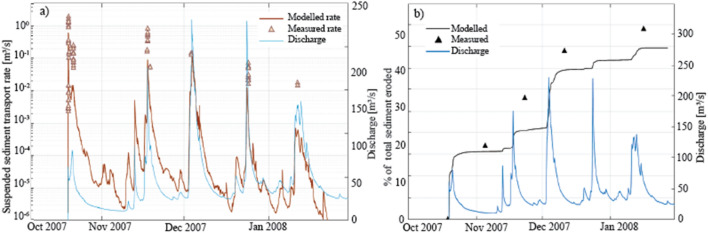
Modelled and measured (**a**) suspended sediment transport rates in the Sandy River at the Marmot Dam Station during cofferdam breaching (results of model calibration and validation) and, (**b**) percentage of sediment eroded from the reservoir during the validation period. The blue line indicates the incoming discharge.

The Reservoir model is updated and re-calibrated based on the measured sediment transport data collected by USGS (see appendix in Major et al.^9^). These data are collected during the first five days after dam removal, during the major erosive process of the cofferdam breaching. The results are: a fall velocity of 0.036 m/s, a critical bed shear stress for erosion of 1 N/m^2^ and an erosion coefficient of 4.17 × 10^−3^ kg/m^2^/s for the sand fraction. The insertion of a wash load layer with a concentration of 0.5 kg/m^2^ along a distance of 500 m upstream of dam in the reservoir water surface provides the best representation of the suspended sediment flux at the time of breaching. The suspended sediment flux reached the value of 0.6 m^3^/s, nevertheless, the computed values remain lower than the measured ones (Fig. 5a). Further details on this re-calibration are given by Panthi^75^.

Validation is based on the cumulative sediment volumes eroded from the reservoir that is measured by USGS^9^ after cofferdam breaching. The computed values are in good agreement with the measured ones (Fig. 5b): after three months, 45% of the total reservoir sediment is eroded whereas the modelled erosion is 40%.

### Suspended sediment outputs during flushing

**Figure 6:**
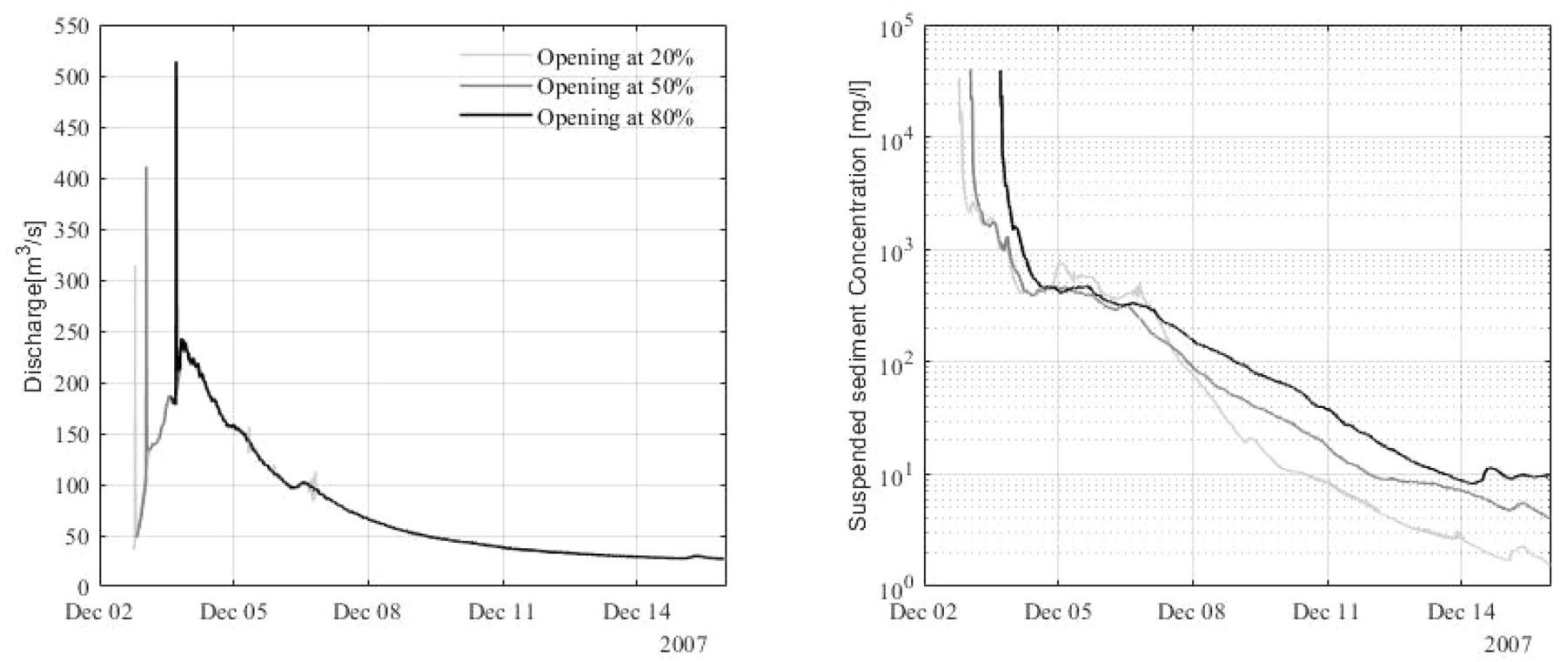
Computed discharge and suspended sediment concentration (sand) for different flushing scenarios corresponding to gate opening at 20%, 50% and 80% of the peak flow, 200 m downstream of the Marmot Dam location.

The sudden opening of the bottom gates results in elevated levels of SSC, but for a relatively short duration (minutes/hours). The highest flushing discharge is 513 m^3^/s, obtained for gate opening at 80% of the peak flow (Fig. 6). The highest sand transport rates are 4.75 m^3^/s, 3.68 m^3^/s and 2.00 m^3^/s for gate opening at 80%, 50% and 20% of the peak flow, respectively. The highest concentrations, however, are rather similar, with 39,000 mg/l, 40,000 mg/l and 33,000 mg/l, respectively (Fig. 6).

The results of the sensitivity analysis, considering different sizes of the fine sediment fraction, show that deposition of silt and clay occurs sparsely in the study area (see Supplementary Fig. A2). This is due to the low fall velocity of these sediment types and the high velocity of the water flow. Instead, sand settles at several locations, mostly between the location of the dam and the entrance of the Sandy River gorge (upper reach, Fig. 1), but also in the pools within the gorge. Due to deposition, sand presents a decrease in concentration in the downstream direction, whereas silt and clay, instead, present an increase due to the entrainment of particles from the riverbed and banks. These fine particles are found to only alter the suspended sediment concentration and hardly change the bed composition. Therefore, the analysis of the effects of flushing on salmon spawning habitats concentrates on sand.

The sand that immediately settles in the upper reach, between the dam location and the gorge, is later transported away by the later flow. This results in persisting high sand concentrations in the downstream reaches. The river banks were eroded in the first few hours of flushing, but later bank erosion stopped as the discharge decreased hence decreasing proximal sediment source.

### Impact of suspended sediment concentration on Chinook salmon

The results of the three flushing scenarios and of the corresponding natural flood are post-processed to determine the corresponding value of severity (Eqs. 1–3) with the aim to quantify the impact of SSC on Chinook salmon, distinguishing the different life stages. The sediment concentration distribution and its duration at different locations along the Sandy River were computed by the River model. The upstream inputs were computed by the Reservoir model, but only for the flushing scenarios. The reference value of the severity index is obtained considering the value of SSC that exceeds 50% in the duration curve, multiplied by the duration of the entire flushing period (Table 3). Table 3 shows lethal conditions for eggs and larvae with values ranging from 10 to 12 indicating up to 40% mortality^35^ for all scenarios and along the entire Sandy River. Increasing order of severity is observed in the downstream direction. The severity is comparatively higher for the flushing scenario with gate opening at 20% of the incoming flood peak, whereas the lowest severity is found for the corresponding natural flood. Note that this analysis is performed for sand. The computed values of SSC for silt and clay are even higher, resulting in greater severity indexes.

**Table 3:** Reference value of severity relative to three life stages of salmon for the considered flushing scenarios and the natural flood scenario at different locations along the Sandy River downstream of the Marmot Dam.

Distance from the dam [km]	Natural Flood scenario			Flushing scenario Gate opening at 20%			Flushing scenario Gate opening at 50%			Flushing scenario Gate opening at 80%		
	Adult	Juvenile	Egg	Adult	Juvenile	Egg	Adult	Juvenile	Egg	Adult	Juvenile	Egg
0.2	*6.79*	*6.96*	**10.85**	*7.48*	*7.61*	**11.13**	*7.52*	*7.64*	**11.14**	*7.78*	*7.89*	**11.25**
4.7	*8.08*	*8.18*	**11.38**	*8.78*	*8.84*	**11.67**	*8.48*	*8.55*	**11.54**	*8.74*	*8.80*	**11.65**
9.2	*8.37*	*8.45*	**11.50**	*8.96*	**9.01**	**11.74**	*8.63*	*8.69*	**11.60**	*8.73*	*8.79*	**11.64**
10.5	*8.52*	*8.59*	**11.56**	*8.99*	**9.04**	**11.75**	*8.85*	*8.90*	**11.69**	*8.90*	*8.95*	**11.72**
15.7	**9.23**	**9.26**	**11.85**	**9.32**	**9.34**	**11.89**	**9.35**	**9.38**	**11.90**	**9.35**	**9.37**	**11.90**
18.0	**9.27**	**9.30**	**11.87**	**9.37**	**9.39**	**11.91**	**9.40**	**9.43**	**11.92**	**9.36**	**9.39**	**11.91**

Egg refers to egg and larvae. The numbers in bold fall within the lethal range, whereas numbers in italics refer to sub-lethal range.

**Figure 7:**
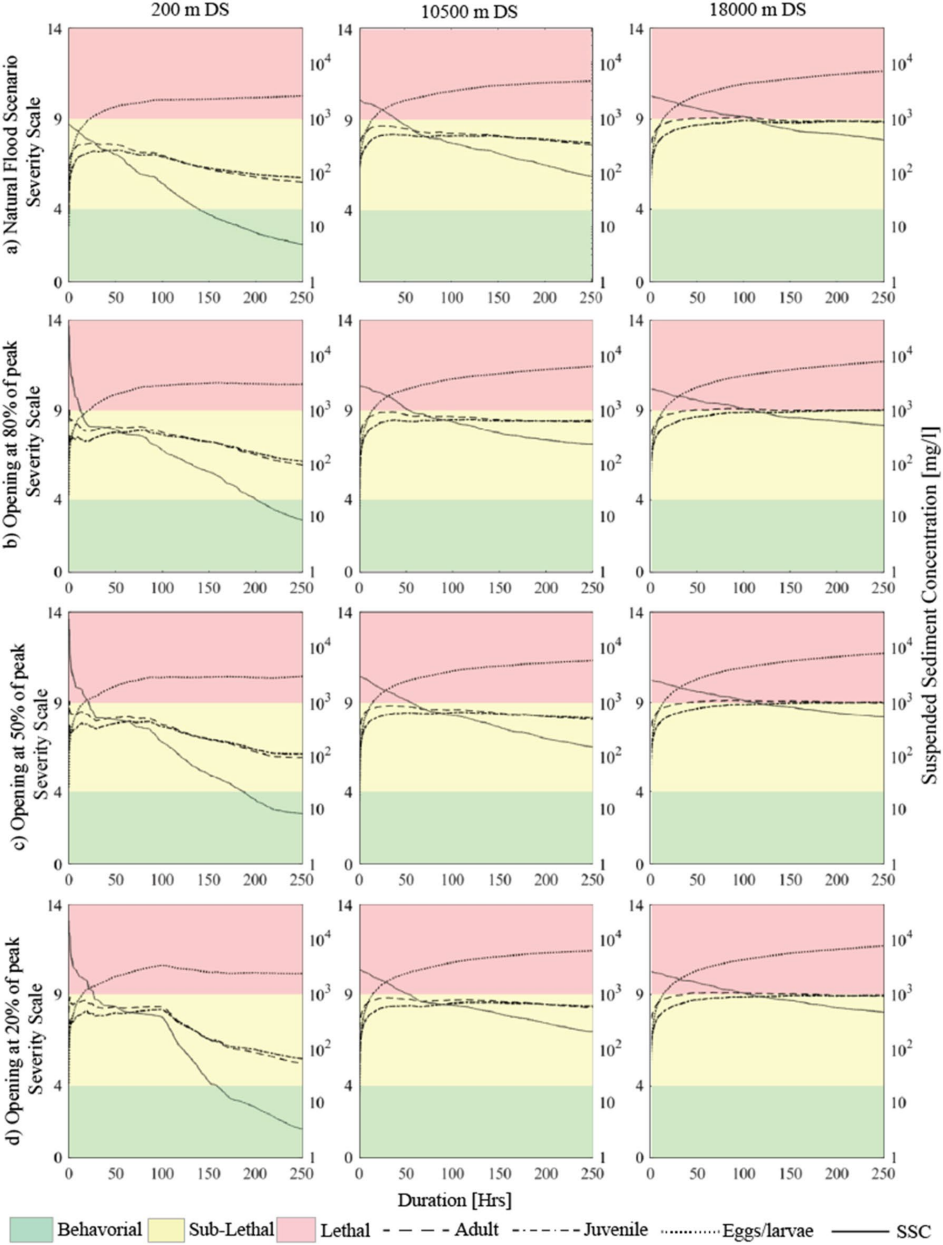
Instantaneous severity for three flushing scenarios at different locations: just downstream of the dam (200 m), at the end of the river gorge (10,500 m) and just before the confluence of the Bull Run River (18,000 m) (**a**) during the corresponding natural flood event; (**b**) gate opening at 80% of incoming peak, (**c**) gate opening at 50% of incoming peak and (**d**) gate opening at 20% of incoming peak.

The time series of severity for the three life stages of salmon is derived from the duration curves (Fig. 7). The results show lethal conditions for egg and larvae for all scenarios, including the corresponding natural flood. Low concentrations are present for longer periods of time and exceed their acceptable duration limit. Instead, the adult and juvenile stages of salmon do not reach the mortality level (SEV > 10).

For the natural flood, the SSC does not exceed the value of 2500 mg/l, whereas for the flushing operations the concentration is as high as 40,000 mg/l.

For the flushing scenario starting at 20% of the flood peak, the suspended sediment concentrations have the longest duration of exposure and produce the highest values of severity. The scenarios with gate opening at 50% and 80% of the flood peak have similar impacts. However, the scenario with the operation starting at 80% of the flood peak releases higher sediment volumes from the reservoir with similar or less acute stress to salmon and for this it is regarded as the most effective flushing operation.

### Impact of sediment deposition on Chinook salmon spawning habitat

**Figure 8:**
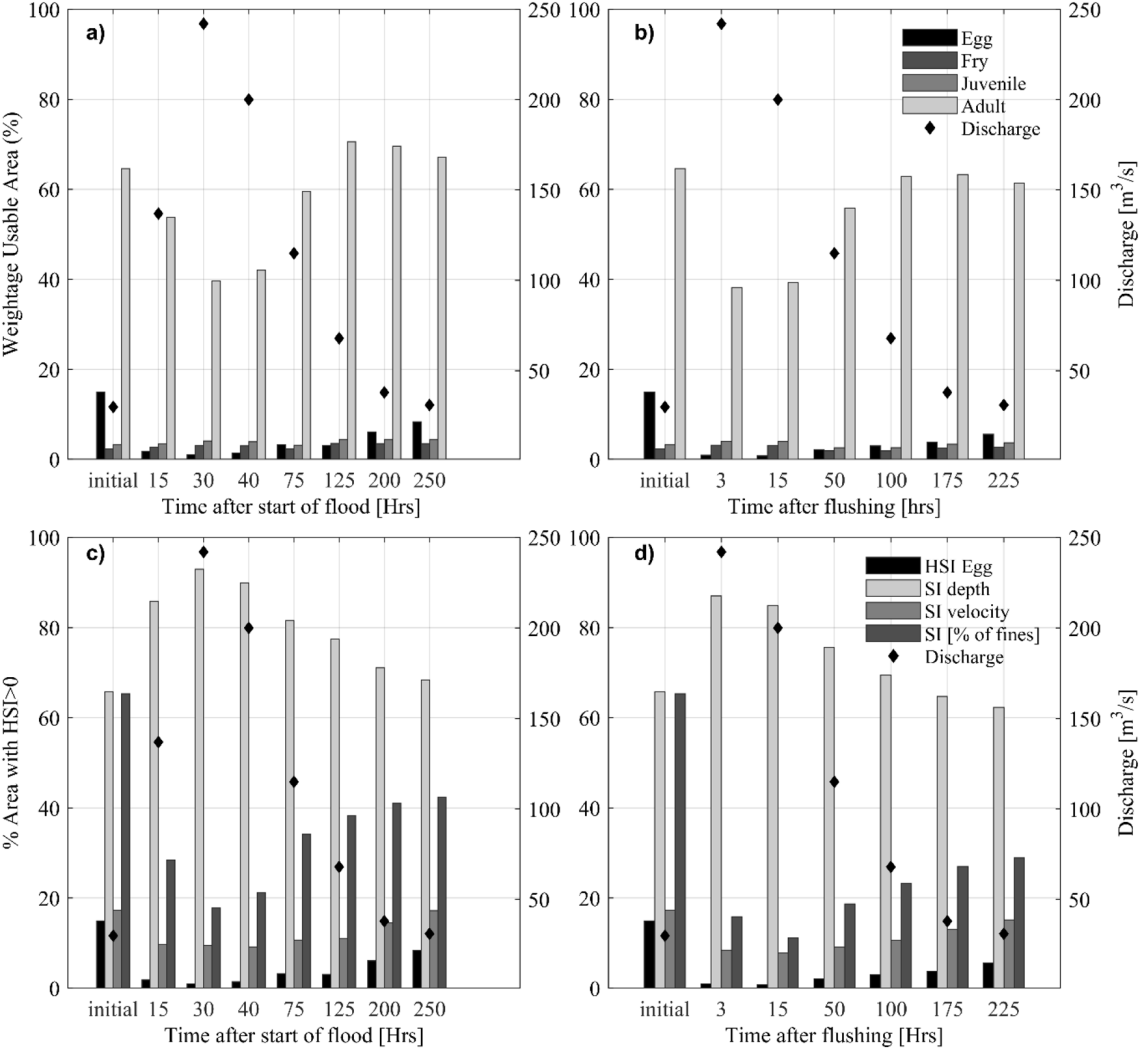
Temporal variation of the percentage of river surface that is suitable for the different life stages of Chinook salmon after the start of (**a**) the natural flood event and (**b**) the flushing operation starting at 80% of the flood peak and, the percentage of area suitable for eggs according to water depth, flow velocity and fine sediment and composite Habitat Suitability (HSI) at different times after the start of (**c**) the natural flood event and (**d**) the flushing operation starting at 80% of the flood peak.

Being the most effective flushing scenario, for the study of the impact on spawning grounds and other habitat areas, we only consider the operation that starts at 80% of the flood peak. The changes in habitat suitability caused by this operation and by the corresponding natural flood at different times are shown in Fig. 8a, b. The foregoing weighted usable areas (Eq. 5) for spawning in the Sandy River are indicated too, for sake of comparison.

Figure 8c, d shows the contribution of water depth, flow velocity and percentage of fines in the substrate through their specific suitability indices for eggs (Eq. 4). The initial condition at time t = 0 h presents a relatively low flow velocity and a pre-flushed riverbed composition. This corresponds to higher suitability for adults with optimum flow velocity. The initial riverbed composition appears less suitable, especially for juvenile fry and egg. After the events, the habitat suitability for salmon at the study area is reduced by both the natural flood and the flushing operation. The reduction in suitable area for adult salmon is due to flow velocity, which depends on discharge and is thus not permanent, whereas for the eggs it is due to the deposition of fines. The highest content of fines in the riverbed occurs 12 h after the discharge peak (Fig. 8c, d). Part of the deposited fine sediment is eroded when the incoming suspended sediment concentration is reduced. At the end of the simulation, when the discharge reaches to baseflow, the usable area for spawning is reduced to 8.3% and 5.6% of the total wet area by the natural flood and the flushing operation, respectively, compared to an initial 15%. This corresponds to a loss of 44% and 62%, respectively.

The comparison between the natural flood and the flushing operation (Fig. 8c, d) shows that after the natural flood the substrate presents a slightly higher recovery with less fine sediment content in the riverbed.

**Figure 9:**
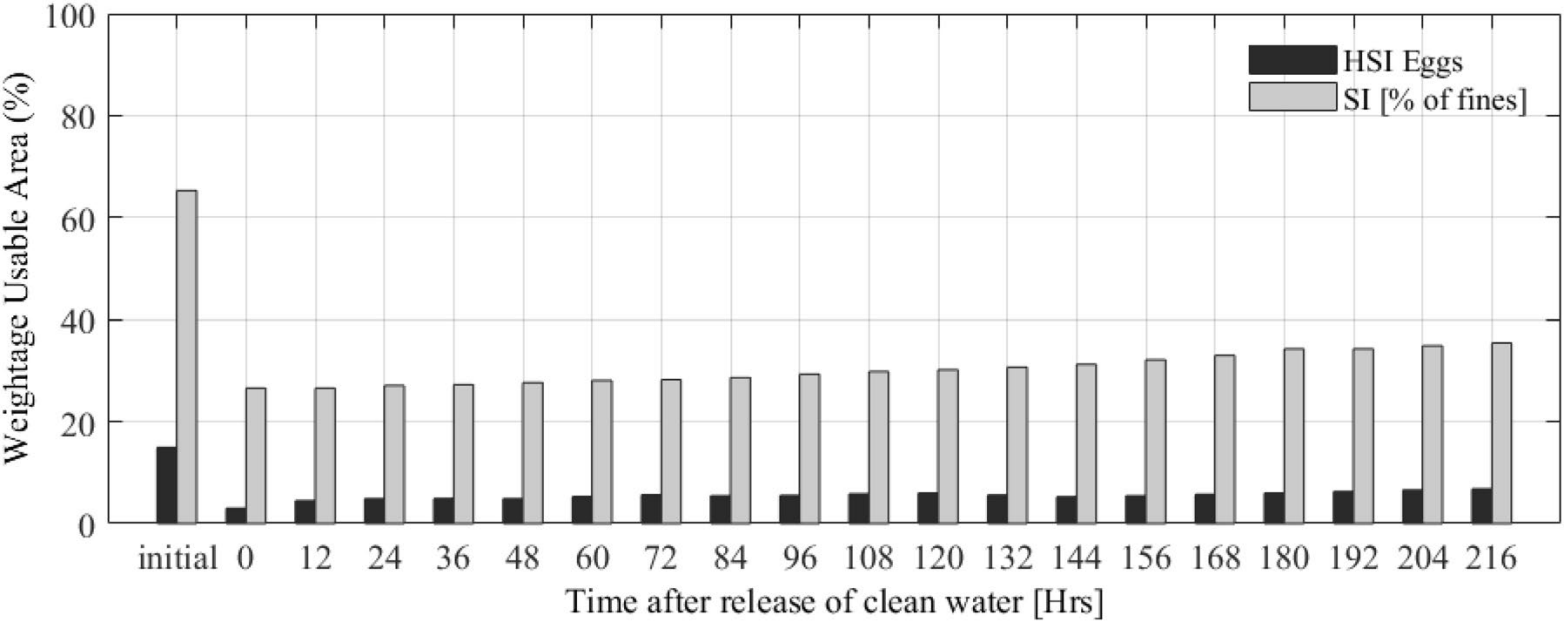
Temporal variation of spawning Weighted Usable Area (WUA) for Chinook salmon (black bars) and suitability in terms of % of fines deposited (grey bars) in the Sandy River with clear water release at a rate of 30 m^3^/s after the flushing operation. The WUA is based on the percentage of fines in the riverbed. “Initial” refers to the conditions just before the flushing operation.

To investigate the possibility to clean the riverbed from the sand deposited during the flushing operation, an additional run is carried out in which the flushing scenario with gate opening at 80% of the flood peak is followed by bottom gate closure and release of clear water to the river at a rate of 30 m^3^/s, which represents the base-flow of the hydrograph (see Supplementary Fig. A1). As clear water flows, fine sediment is progressively removed from the riverbed. After nine days of clear water release, however, the riverbed is still only slightly cleaned (Fig. 9). This shows that at the chosen flow rate the riverbed takes weeks to be cleaned up and indicates that flushing would cause a long-term disturbance to the spawning habitats.

## Discussion

The concentration of suspended sediment is key to salmon survival. However, the results indicate that the magnitude of the suspended sediment release can be underestimated by the Reservoir model. This model represents well the overall phenomena, with rapid channel excavation within the reservoir deposit and retrogressive bed erosion. The widening of the excavated channel, though, is small compared to the measured one and might be the reason for the underestimation of sediment release. However, this might be also related to the uncertainty posed by the composition of the deposit layers and horizontal sediment sorting in the reservoir. The possible underestimation of the sediment releases from the reservoir should be always considered when analyzing the results of the model, particularly if the predicted concentrations fall just under the sub-lethal threshold.

About the downstream river, the availability of fine sediment in the bed is important for the computation of both SSC and deposition rates by the River model. The initial bed composition was obtained in a straightforward way through the spin-up run, but without the possibility to calibrate the morphodynamic part of the model. Lack of field data meant uncertainty in the identification of the initial spawning ground suitability, i.e. before the flushing operations and the natural event, affecting the assessment of their impacts. The difficulty of establishing the initial riverbed composition affected the impact assessment even more, considering that the results of the River model show that sediment entrainment from the riverbed and eroding banks^87^ is important for the assessment of both stress and habitat losses (Fig. 7).

About the stress caused by the exposure to suspended solids, the results show that the differences in severity between the flushing scenarios and the natural flood are small. Flushing operations produced higher suspended sediment concentrations but for shorter periods of time compared to the natural flood and for this they resulted in similar severity levels. For instance, the conditions become lethal for eggs and larvae in all cases and the natural flood conditions result only in slightly lower severity for adults and juveniles as compared to the flushing operations. The deposition of fine sediment on the riverbed caused a similar reduction of suitable spawning habitat, but the recovery was higher and faster at the end of the flood event compared to the flushing. The severity was found to increase in downstream direction, due to the rise of suspended sediment concentration caused by the entrainment of fines from the riverbed, and the eroding banks.

The severity for eggs and larvae mainly depends on the duration of exposure to suspended solids. The analysis is based on the severity scale of Newcombe and Jensen^35^, but this might not well represent the effects. This scale does not consider the stage or condition of the fish before the exposure, and the severity, calculated with one value of the concentration, does not consider the cumulative effect of exposure. Moreover, the derived severity scale is based on field data analysis, whereas the response to suspended solids is highly site-specific^10^. For instance, the fish that is exposed to extreme events frequently can be more tolerant or able to escape to neighboring tributaries with lower SSC^88^.

Next to stress severity, the deposition of fines on the riverbed during the events is found to produce an important loss of spawning habitats. The results of the River model indicate that up to 95% of the eggs laid in the riverbed would be buried. Lab experiments by Jensen et al.^24^ showed a decrease in egg and fry survival by 16.9% for every 1% increase in the content of fines (< 0.85 mm) in the substrate.

It is important to note that the highest natural floods of the Sandy River normally occur in November, December and January, followed by lower flow peaks in February-March^50,51^. In this river, salmon spawns in September-December. This means some natural floods occur during the critical spawning and egg incubation periods. A decline in live fish and redd counts after the rain storms of October 2016 was reported^58^, indicating that the water was highly turbid, qualitatively confirming the results of this study.

**Figure 10:**
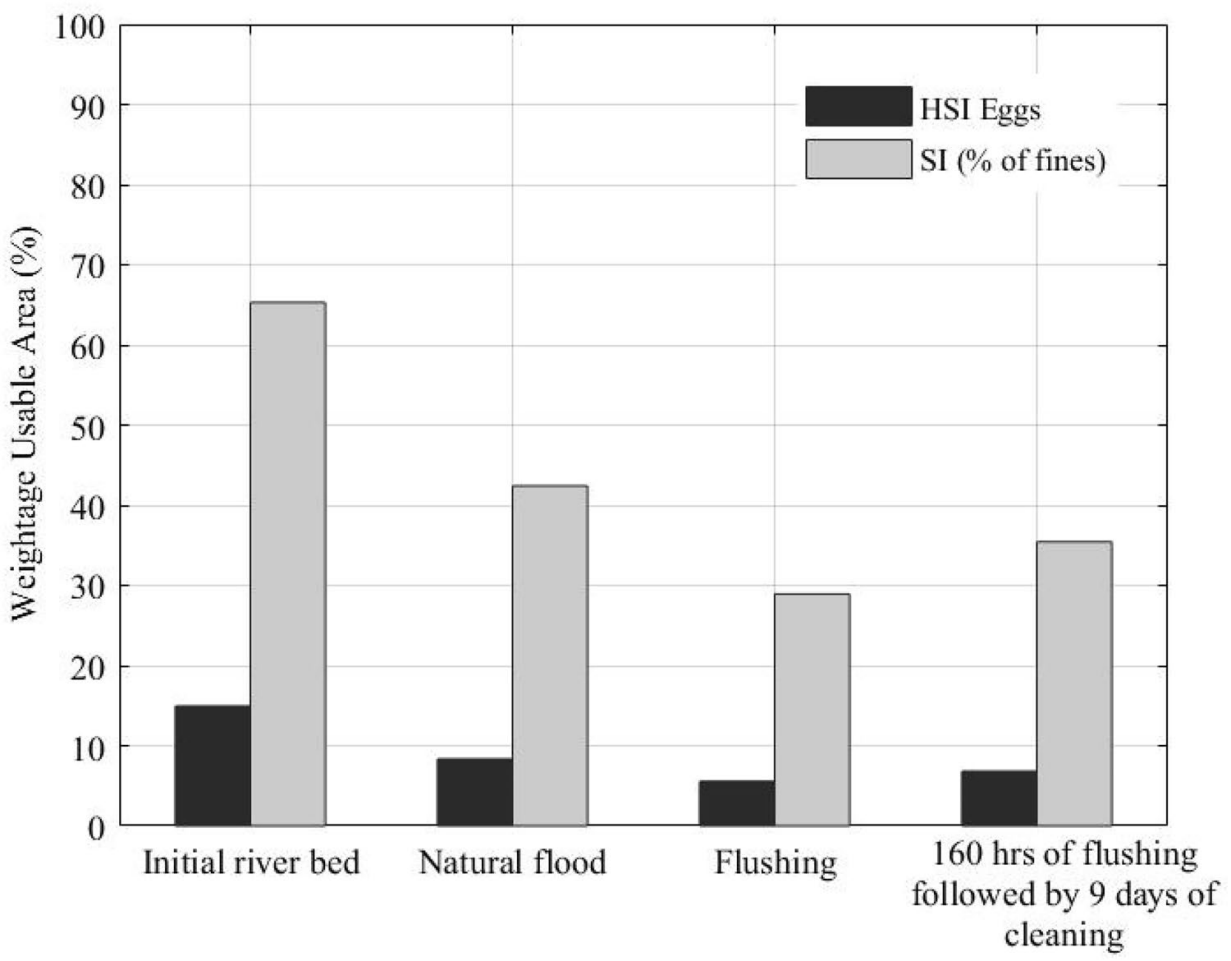
Comparison of suitable spawning areas of the riverbed with natural flood, flushing only, and flushing followed by clean water release at a constant rate equal to the river base-flow. “Suitability” assumes that gravel of the right size is present in the riverbed.

The comparison between spawning area suitability shows that flushing causes a more persisting disturbance to spawning habitats than the natural flood, even if followed by riverbed cleaning by the constant release of clear water at a rate equal to the river base-flow (Fig. 10). The impact is present for almost a year if the SSC of the flow is high^89^, slowing the river recovery process and any restoration plans^90^. A more irregular clear water release, presenting short but higher discharge peaks, could increase the riverbed flushing rate and reduce the habitat restoration times. In any case, releasing large amounts of clear water might reduce the water storage in the reservoir to unacceptable levels, which limits the applicability of this type of operations. This means that riverbed flushing requires a specific study leading to the optimization of the release of precious clear water from the reservoir.

It is necessary to finally consider that the analysis of habitat suitability is only based on flow velocity, water depth and percentage of fines. The study considered a specific flood event, the equivalent one, i.e. the one considered also for the design of the flushing operation. Considering another flood event for both the flushing operations and the natural flood would lead to different physical conditions and habitat alterations. It is important to consider that also other factors, such as temperature, dissolved oxygen, turbidity, season, and water quality, as well as multiple events, which might affect the results, are not considered in this study.

Extreme events change not only the sediment composition, but also the riverbed topography and the river course, which might alter the connectivity of the floodplains and hence the riverine habitats^90,91^. This study does not consider this aspect. Being based on models (severity and HSI) with high degrees of uncertainty, the results of this study should be interpreted in terms of comparison between scenarios.

Finally, it should be considered that the results of this study are case-sensitive, with specific availability and type of sediment in the reservoir, flushing discharge, and reservoir characteristics, all factors that govern the quantity and quality of the sediment released from the reservoir and its impact on salmon.

## Conclusions

This work investigates the short (acute stress) and the long-term effects (river habitat alterations) of sediment flushing from a reservoir on Chinook salmon. Considering that flushing operations and natural floods present many similarities, the aim of this study is to assess the difference in terms of impacts between reservoir flushing and natural floods. Can the negative effects be reduced by proper planning/design of flushing operations?

To meet the goal, the effects of reservoir flushing, conveniently starting during a flood event, are compared to the effects of the corresponding natural flood wave without reservoir. The Sandy River, Oregon USA, was chosen as a case study due to the availability of measured data from the period around the Marmot Dam removal in 2007, considering dam removal as an extreme case of sediment flushing. A 2D morphological model was used to simulate the propagation and deposition of fine sediment along the river downstream of the dam. The sediment inputs generated by the flushing operations were simulated by means of another 2D model covering the Marmot Dam Reservoir and the dam, assumed to be in operation with appropriate bottom gates.

The model results allowed assessing the stress caused by the exposure to suspended solids (short-term effect) through the analysis of severity indices derived for salmonids and the habitat loss (long-term effect) through the analysis of Habitat Suitability Indexe for Chinook salmon.

The results indicate that excessive exposure to suspended sediment concentration during either the natural or the artificial (flushing) high-flow events is lethal to salmon eggs and alevins. Spawning habitat losses for flushing and flood events are found to be very similar, with 95% and 93% of losses, respectively. The eggs already laid would have severe physiological and lethal effects during a flushing operation, as well as during a natural flood.

Although the short-term impacts are similar, the long-term impact on spawning grounds due to fine sediment deposition is found to be higher in case of flushing. Cleaning of the riverbed by releasing sediment-free water at a rate equal to the river base-flow appears not effective in gaining suitable spawning beds and reaching the conditions that are present after the natural flood. The effectiveness of riverbed cleaning with different clear-water flow releases, possibly including peak discharges of short duration mimicking natural flows, should be further investigated.

Reservoir flushing with gate opening at 80% of an incoming flood peak is advisable, not only considering the efficiency of the operation in terms of sediment release and duration, but also because this is the flushing operation having the smallest impact, although only slightly.

In the study area the highest flows normally occur in November-January whereas the critical spawning and egg incubation period of salmon is September to December. Fry appear approximately four months after eggs are laid, i.e. in December-March. This means that most natural floods harm salmon eggs and alevins, as the results of this study show, especially if they occur in the last months of the year. Considering this, the best moment for sediment flushing would be after March. However, the operation should start at the peak of a flood wave and subsequent riverbed cleaning operations ideally need natural floods too. Looking at the typical yearly hydrograph of the Sandy River, having this operation in the second half of January could offer an acceptable compromise between loosing eggs and recently hatched fish and need to flush the reservoir and clean the riverbed.

If flushing starts at the peak of a flood wave with duration comparable to the natural event, the timing of the operation would fall in the temporal ranges of natural floods, to which the river biota has adapted. By showing that the effects of floods and flushing are comparable, at least with regard to salmon, the results of this study indicate that well-planned timing of flushing followed by effective river bed cleaning, if achievable, would substantially minimize the effects of dam operation.

## Supplementary Information

Supplementary Information.

## Data Availability

All data, model inputs and files are downloadable upon request to the corresponding author from: http://www.hydroshare.org/resource/7674e96fa50b436fba80ba59d566a767. Information on discharge data and sediment data can be obtained from: https://pubs.usgs.gov/pp/1792/ and USGS stations.
