# Supplementary material for: Effects of sediment flushing operations versus natural floods on Chinook salmon survival
Source: Sci Rep. 2022 Sep 12;12:15354. doi: 10.1038/s41598-022-19294-2 (PMC9467979; doi:10.1038/s41598-022-19294-2)
Supplement: Supplementary file 1 — Supplementary Information. [file 41598_2022_19294_MOESM1_ESM.docx]

# **Supplementary Information for**

Effects of sediment flushing operations versus natural floods on Chinook salmon survival

Manisha Panthi^1,2,*^, Aaron A. Lee^3^, Sudesh Dahal^4^, Amgad Omer^5^, Mário J. Franca^1,6,7^, Alessandra Crosato^7,8^

^*^corresponding author: [manisha.panthi@usu.edu](mailto:manisha.panthi@usu.edu)

Table A1 Characteristics of suspended solids for the scenarios of the sensitivity analysis.

| Serial Number | Suspended Solid | Diameter [mm] | Fall Velocity [m/s] | Input Boundary Condition |
| --- | --- | --- | --- | --- |
| 1 | Sand | 0.3 | 0.036 | Output from Reservoir model RF_80 |
| 2 | Silt | 0.063-0.03 | 0.0008 |  |
| 3 | Clay | <0.002 | 0.000003 |  |


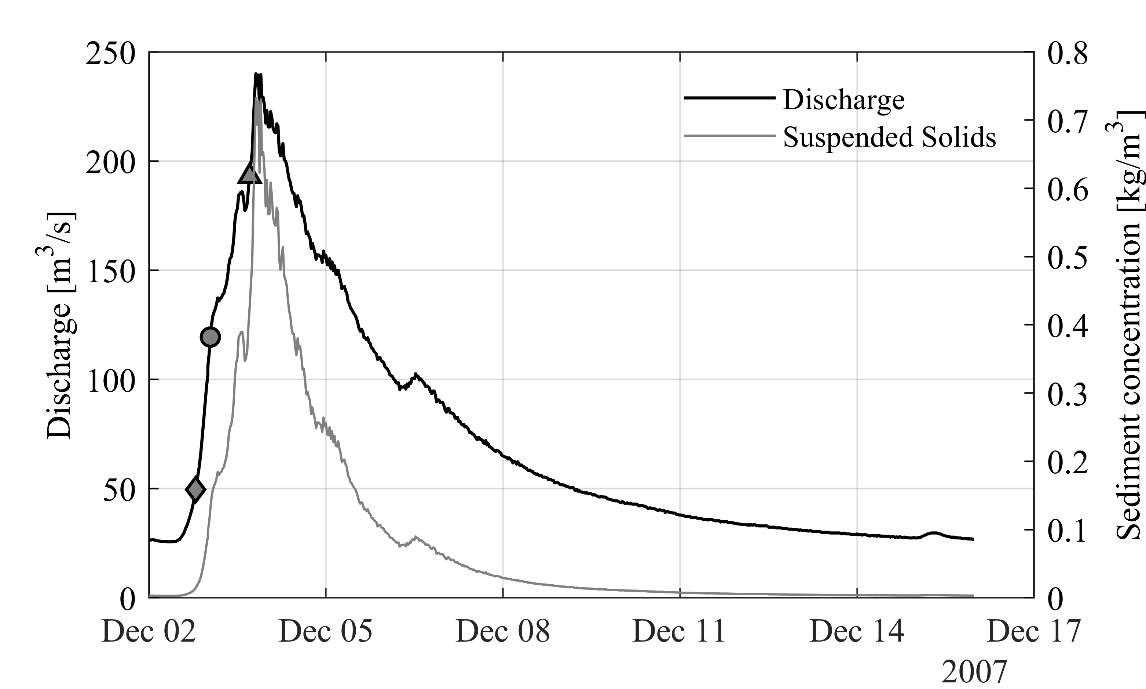


Figure A1 Inflow discharge and suspended solids concentration for natural flood and flushing scenarios. The little diamond, circle and triangle on the discharge hydrograph indicate the moment of gate opening for scenario RF_20, RF_50 and RF_80 of Table 1, respectively.


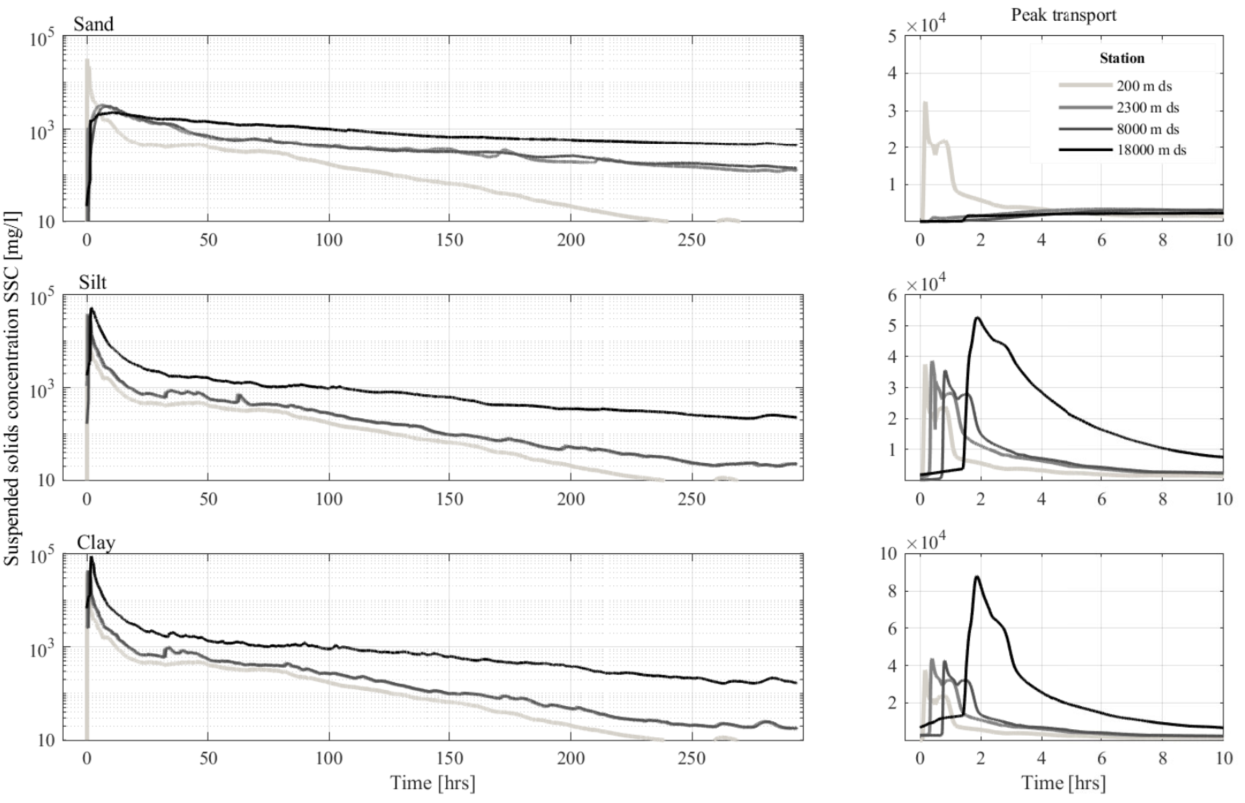


Figure A2 Temporal variation of concentration of sand, silt and clay in the Sandy River 200 m downstream of the dam location (light grey line), at the start of the gorge, 2,300 m (grey line), after the end of gorge 8,000 m (dark grey line) and before the confluence of the Bull Run River 18,000 m (black line). The left panels cover the entire simulation period of 300 hours whereas the right panels focus on the first 10 hours.
